# The lncRNA HOTAIR transcription is controlled by HNF4α-induced chromatin topology modulation

**DOI:** 10.1038/s41418-018-0170-z

**Published:** 2018-08-28

**Authors:** Cecilia Battistelli, Giovanna Sabarese, Laura Santangelo, Claudia Montaldo, Frank J. Gonzalez, Marco Tripodi, Carla Cicchini

**Affiliations:** 1 Istituto Pasteur-Fondazione Cenci Bolognetti, Department of Cellular Biotechnologies and Haematology, Sapienza University of Rome, Rome, Italy; 2 National Institute for Infectious Diseases L. Spallanzani, IRCCS, Rome, Italy; 3 Laboratory of Metabolism, Center for Cancer Research, National Cancer Institute, National Institutes of Health, Bethesda, MD USA

**Keywords:** Epigenetics, Oncogenes

## Abstract

The expression of the long noncoding RNA HOTAIR (*H*OX *T*ranscript *A*ntisense *I*ntergenic *R*NA) is largely deregulated in epithelial cancers and positively correlates with poor prognosis and progression of hepatocellular carcinoma and gastrointestinal cancers. Furthermore, functional studies revealed a pivotal role for HOTAIR in the epithelial-to-mesenchymal transition, as this RNA is causal for the repressive activity of the master factor SNAIL on epithelial genes. Despite the proven oncogenic role of HOTAIR, its transcriptional regulation is still poorly understood. Here hepatocyte nuclear factor 4-α (HNF4α), as inducer of epithelial differentiation, was demonstrated to directly repress HOTAIR transcription in the mesenchymal-to epithelial transition. Mechanistically, HNF4α was found to cause the release of a chromatin loop on HOTAIR regulatory elements thus exerting an enhancer-blocking activity.

## Introduction

The lncRNA HOTAIR (*H*OX *T*ranscript *A*ntisense *I*ntergenic *R*NA [1],) is a transcript, antisense to the mammalian HOXC (homeobox transcription factor C) locus, that is largely deregulated in cancer. HOTAIR transcription positively correlates with poor prognosis and progression in several epithelial tumors, including hepatocellular carcinoma (HCC) and gastrointestinal cancers [2–6]. Consistently, forced expression of lncRNA HOTAIR in epithelial cancer cells causes the acquisition of metastatic properties [2, 4, 5], whereas its knockdown significantly impairs migratory and invasive properties of cells [4]. HOTAIR acts as an assembling scaffold for the Polycomb member EZH2, main writer of repressive histone marks, and therefore it is involved in the targeting of H3K27 methylation to target regions of the genome. Notably, this role impacts epithelial cell reprogramming in both physiology and pathology [2, 7, 8].

Reprogramming of epithelial cells relies on the transdifferentiation processes, known as epithelial-to-mesenchymal transition (EMT), and the reverse mesenchymal-to-epithelial transition (MET). EMT/MET plasticity is essential for organogenesis, development, wound healing, and regeneration, and is aberrantly activated in fibrosis, tumor progression, and metastasis [9]. We recently demonstrated that HOTAIR expression is induced in hepatocytes undergoing EMT and functions to bridge, in specific chromatin sites, the interaction between EZH2 and Snail. In other words, the EMT “master” factor Snail (i.e., sufficient to trigger and orchestrate the transition) conveys the Polycomb catalytic subunit to specific sites by means of a direct interaction with HOTAIR. Thus, in epithelial transdifferentiation, HOTAIR behaves as a “mesenchymal” gene with a functional role in the Snail-mediated repression of epithelial genes [7, 10].

Despite the strong correlation between HOTAIR expression, EMT, and tumor progression [2, 4, 11–13], studies on the molecular events regulating the transcription of this lncRNA are still limited [14].

Several evidence demonstrated the role of the orphan nuclear receptor hepatocyte nuclear factor 4-α (HNF4α) as master regulator of differentiation and epithelium formation in hepatocytes [15, 16] as well as in colon cells [17–20]. The role of HNF4α in the regulation of the MET [21] and in the maintenance of a stable epithelial phenotype depends on the capacity of this transcriptional factor to act as a direct repressor of both master EMT regulators and mesenchymal genes [22, 23].

Here, HNF4α is identified as a direct transcriptional repressor of the HOTAIR gene in epithelial cells by studying (i) in vitro hepatocyte cells able to undergo EMT/MET dynamics [10, 24–26], (ii) an in vivo model of hepatocyte-specific *Hnf4α* knockout (*Hnf4a*^*F/F;AlbERT2cre*^ mice [27]), and (iii) colon cancer cells representative of different states of tumor progression [28]. Notably, HNF4α-mediated repression in EMT/MET dynamics is associated with chromatin topological remodeling of HOTAIR regulatory sequences. Our data demonstrate that HNF4α binds to HOTAIR regulatory sequences and causes the removal of a chromatin loop including an enhancer, located 150 Kb downstream of the HOTAIR transcriptional start site (TSS), and the proximal promoter [14].

## Experimental procedures

### Cell culture conditions and animal model

Differentiated hepatocyte cells [10, 22, 29] were grown in RPMI 1640 supplemented with 10% fetal bovine serum (FBS) (GIBCO® Life Technology, Monza, Italy), 50 ng/ml EGF, 30 ng/ml IGF II (PeproTech Inc., Rocky Hill, NJ, USA), 10 μg/ml insulin (Roche, Mannheim, Germany), and antibiotics, using collagen I (GIBCO® Life Technology, Monza, Italy) coated dishes. SW480 and SW620 cells [28] were grown in DMEM (according to Wu et al. [30]) supplemented with 10% FBS (GIBCO® Life Technology, Monza, Italy), and antibiotics. Where reported, differentiated hepatocytes and SW620 were treated with 5 µM TGFβ1 (PeproTech Inc., Rocky Hill, NJ, USA), respectively, for 24 and 72 h. Where indicated, the cells were infected with a retrovirus expressing human HNF4α2 (pLPCX HNF4α2) or the empty vector (pLPCX), as a control [24]. To produce recombinant retroviruses, 293GP packaging cells were transiently transfected according to standard procedures with the HNF4α retroviral construct together with the VSV envelope protein encoding plasmid. Viral particles were collected 48 h after transfection. Cells were collected 48 h after retroviral infection for further analyses.

*Hnf4α*^*F/F;AlbERT2cre*^ mice were previously described [26, 27]. Animal studies were performed according to the guidelines and approval of the National Cancer Institute, National Institutes of Health, Animal Care and Use Committee, as previously reported in [26].

### siRNA interference

Cells were transfected with Lipofectamine 2000 reagent (Invitrogen, San Diego, CA, USA), as in ref. [10], by using equal amounts of small interfering RNA (siRNA), specifically against GFP (5′-GGCUACGUCCAGGAGCGCACC-3′), as control, or human HNF4α [31, 32], murine HNF4α [26], human HOTAIR (5′-GAACGGGAGUACAGAGAGA-3′;5′-UAACAAGACCAGAGAGCUG-3′; 5′-CCACAUGAACGCCCAGAGA-3′). Analyses of RNAs and proteins were performed 48 h after transfection.

### RNA extraction, reverse transcription (RT), and real-time polymerase chain reaction (RT-qPCR)

Total RNAs were obtained from liver samples by TRIzol (Ambion, Life Technology, Monza, Italy) or from cells by RNAeasy Mini Kit (QIAGEN GmbH, Hilden, Germany). Reverse transcription was performed by using the iScriptTM c-DNA Synthesis Kit (Bio-Rad Laboratories, Inc., Hercules, CA, USA) and qPCR reactions on cDNAs by using GoTaq® qPCR Master Mix (Promega, Madison, WI, USA). The relative amounts were obtained by 2^-ΔCt^ method and normalized with respect to the housekeeping gene 18 S (mouse) or L32 (human). The list of primers is shown in Supplementary Table 1.

### Western blot

Cells were lysed in Laemmli buffer, subsequently the proteins were resolved on sodium dodecyl sulfate polyacrylamide gel electrophoresis and transferred to Nitrocellulose membrane 0.45um (162–0115; Bio-Rad Laboratories, Hercules, CA). The following primary antibodies were used for immunoblotting: α-HNF4α (Santa Cruz Biotechnology, Inc., CA), α-Snail (Cell Signaling Technology, Danvers, Massachusetts), α-E-cadherin (BD transduction laboratories, Franklin Lakes, New Jersey), and α-GAPDH (Millipore Corp., Bedford, MA), used as a loading control. The immune complexes were detected with horseradish peroxidase-conjugated species-specific secondary antiserum (Bio-Rad Laboratories, Hercules, CA) then by enhanced chemiluminescence reaction (Pierce, Rockford, IL).

### Chromatin immunoprecipitation (ChIP) analysis

ChIP analysis was performed as reported previously [10] by using 5 μg rabbit α-HNF4α (H-171, sc-8987; Santa Cruz Biotechnology, Inc., CA) or the negative control normal rabbit immunoglobulin (IgG) (Millipore Corp., Bedford, MA). In total, 5 ng of immunoprecipitated DNA and the relative controls were used as templates for real-time qPCR analysis, performed in triplicate. The list of primers is shown in Supplementary Materials. qPCR analysis of the immunoprecipitated samples and of the negative controls (IgG) were both normalized to total chromatin input and expressed as percentage of Input (% Input). Histone ChIP analysis was performed by using 5 µg of the specific antibody (H3K27me3; 07–449; Millipore Corp., Bedford, MA) or of the negative control normal rabbit IgG (Millipore Corp., Bedford, MA), as reported previously [10]. The DNA was extracted with phenol–chloroform, precipitated with ethanol and resuspended in 50 μl of water, then used in the downstream qPCR analyses (primer pairs listed in Supplementary Table 1).

### Chromosome conformation capture

3 C assays were performed as described previously [33, 34]. After chromatin crosslinking and nuclei isolation, DNA was digested overnight with 400 U of EcoRI restriction enzyme and ligated in 1 × ligation buffer (New England Biolabs) for 4 h at 16 °C followed by 30 min at room temperature. Ligation products were extracted with phenol–chloroform, precipitated with sodium acetate and ethanol, washed with 70% (v/v) ethanol, and resuspended in 150 ml of distilled water. As negative controls the not-digested and not-ligated sample was analyzed together with the digested and not-ligated sample.

The primers used for 3 C sample amplifications were reported in Supplementary Table 1. To quantify the amount of DNA in each amplification the primers pair for GAPDH Promoter was used. To quantify the digestion efficiency, the primers pair for HoxC Enhancer, designed on a region containing a single restriction site, was used. The amplification relative to this region was normalized respect to the total amount of DNA (GAPDH promoter). To evaluate the interaction frequency between the distal enhancer and the proximal promoter of HOTAIR the primers pair for HOTAIR gene was used. The amplification signal relative to the interaction frequency was normalized respect to the total amount of DNA (GAPDH promoter) and to the digestion efficiency (HoxC Enhancer).

### Statistical analysis

The *t* test was used for statistical analyses. All the tests were one-tailed and a *p* value < 0.05 was considered statistically significant (* symbol). Data were obtained from independent experiments performed at least in triplicate and expressed as mean ± SEM.

For liver samples analysis the Mann–Whitney test was used. Data were obtained from six wild-type and six knockout mice and expressed as mean ± SEM.

### Computational analysis

The regulatory sequences (up to 1 kb upstream of transcription start site) of murine and human HOTAIR and human E-cadherin were obtained from ENSEMBL (http://www.ensembl.org) and submitted to MatInspector Professional (release 8.0, Genomatix, Munchen, Germany), using the vertebrate matrix library and optimized thresholds, to identify putative HNF4α-binding sites.

## Results

### HOTAIR is required for the Snail-dependent repression of epithelial genes in colon cancer cells

**Fig. 1:**
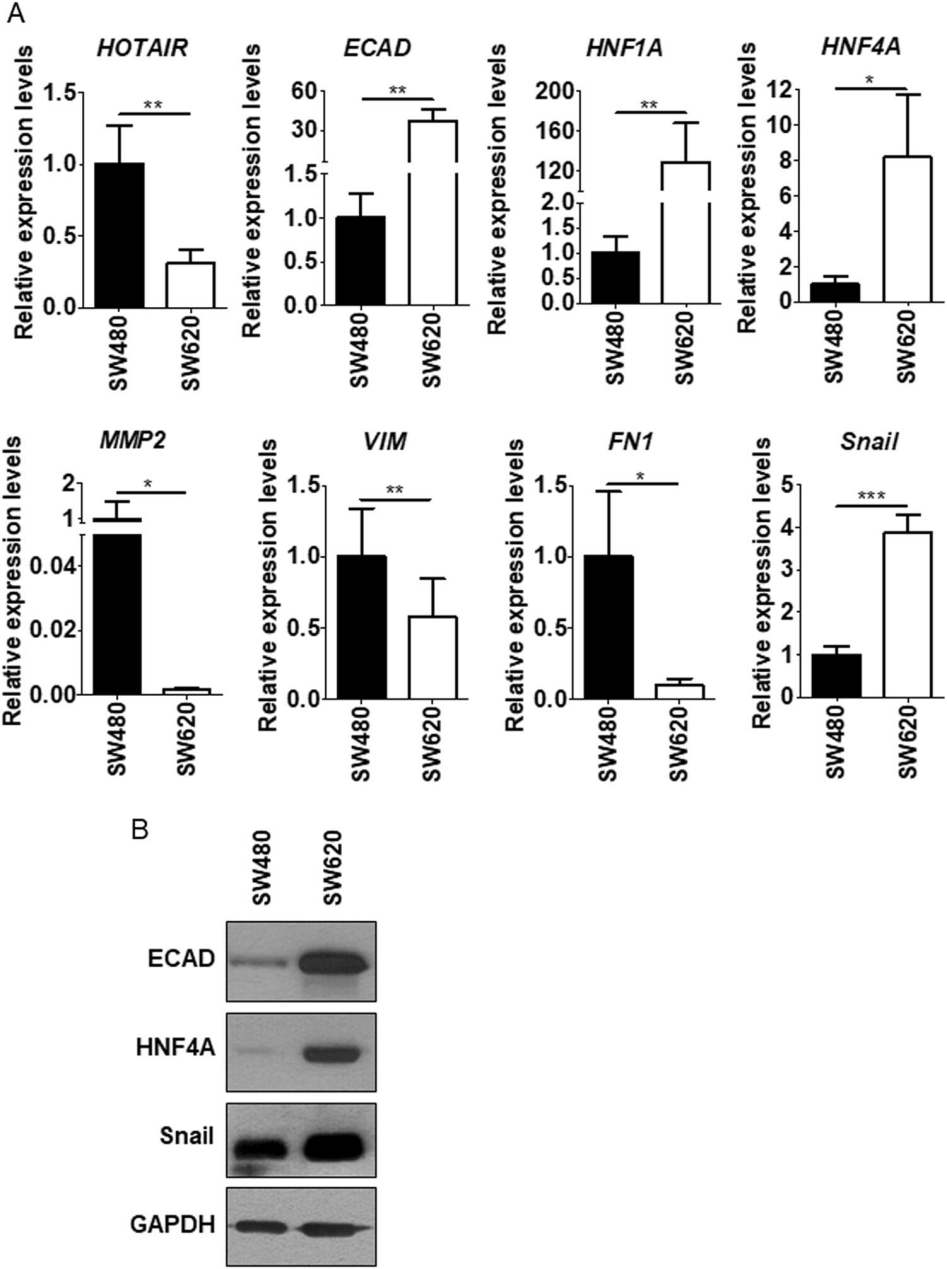
EMT/MET molecular phenotype of SW480 and SW620 cells. **a** RT-qPCR analysis for HOTAIR and for the indicated epithelial (*ECAD, HNF1A, HNF4A*) and mesenchymal (*MMP2, VIM, FN1, Snail*) markers on SW480 and SW620 cells. The values are calculated by the ΔCt method and expressed as relative to the L32 RNA levels and shown as mean ± S.E.M. Statistically significant differences (*p < 0.05; **p < 0.01; ***p < 0.001) are reported for eight independent experiments. **b** Western blot analysis for the indicated markers on protein extracts from cells as in **a**. Protein amount was normalized by immunoblotting for GAPDH, as indicated. All the experiments have been performed in triplicate and WB images represent one indicative experiment of three independent ones

**Fig. 2:**
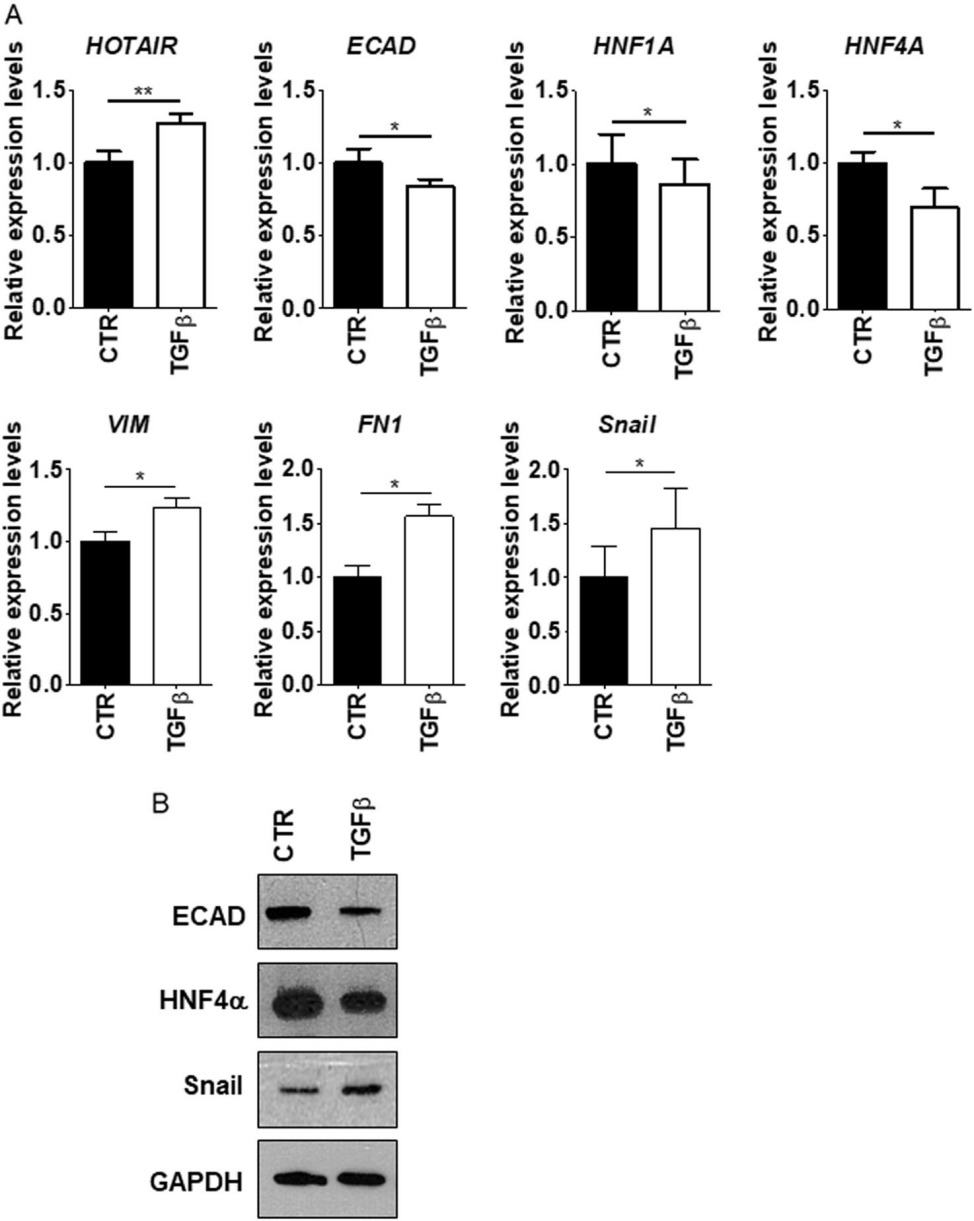
SW620 cells undergo into EMT. **a** RT-qPCR analysis for HOTAIR and for the indicated epithelial (*ECAD, HNF1A, HNF4A*) and mesenchymal (*VIM, FN1, Snail*) markers on SW620 cells treated or not with TGFβ. The values are calculated by the ΔCt method and expressed as relative to the L32 RNA levels and shown as mean ± S.E.M. Statistically significant differences (*p < 0.05; **p < 0.01) are reported for four independent experiments. **b** Western blot analysis for the indicated markers on protein extracts from cells as in **a**. Protein amount was normalized by immunoblotting for GAPDH, as indicated. All the experiments were performed in triplicate and the WB images represented as one indicative experiment of three independent experiments

**Fig. 3:**
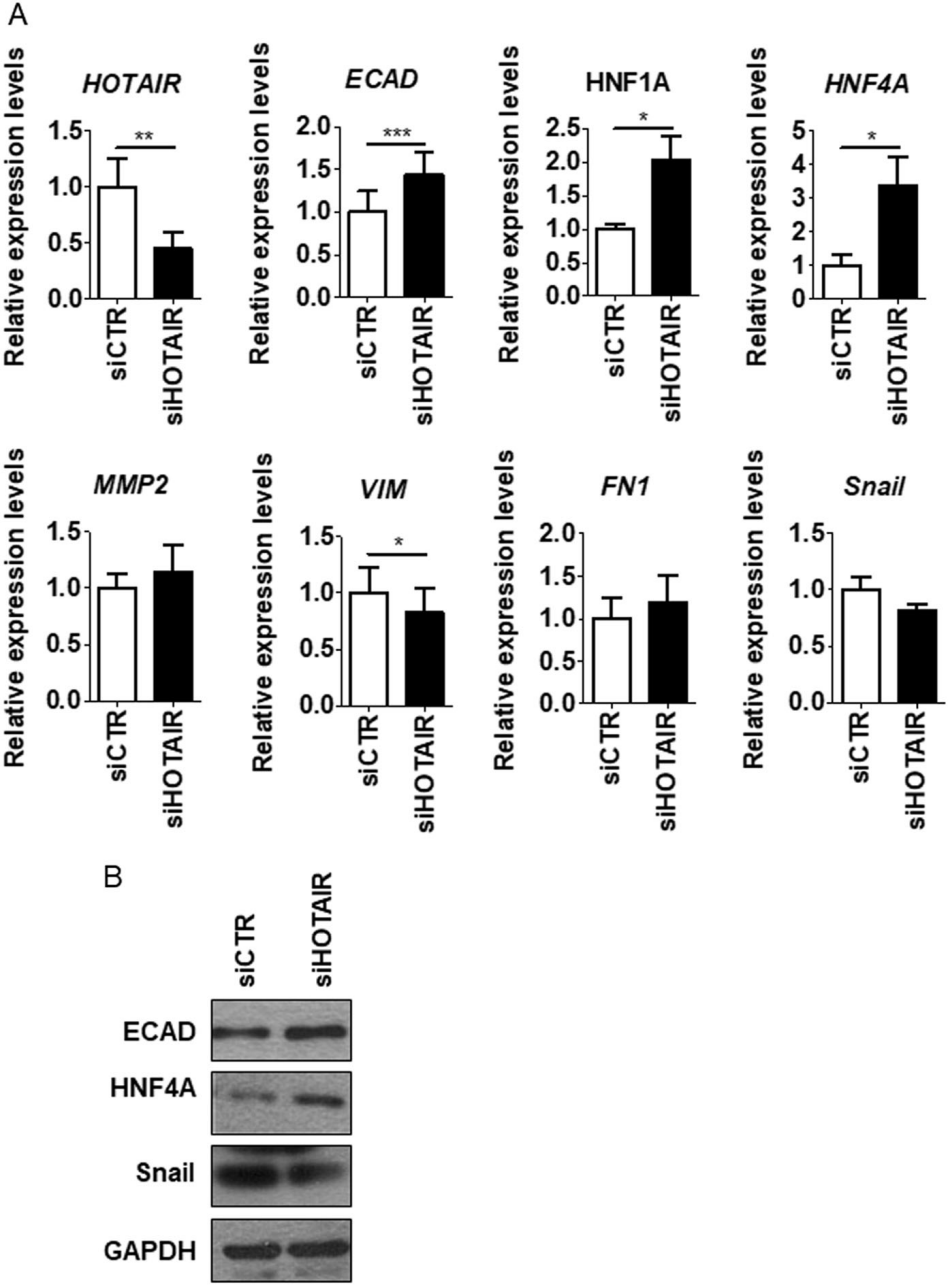
HOTAIR has a functional role in EMT of colon cells. **a** RT-qPCR analysis for the indicated epithelial (*ECAD, HNF1A, HNF4A*) and mesenchymal (*MMP2, VIM, FN1, Snail*) markers in HOTAIR-silenced cells (siHotair) in comparison with siGFP cells (siCtr), as control. The values are calculated by the ΔCt method, expressed as fold of expression vs. the control (arbitrary value = 1) and shown as means ± S.E.M. Statistically significant differences (*p < 0.05; **p < 0.01; ***p < 0.001) are reported for five independent experiments. **b** Western blot analysis for the indicated markers on protein extracts from cells treated as in **a**. Protein amount was normalized by immunoblotting for GAPDH, as indicated. All the experiments have been performed in triplicate and WB images represent one indicative experiment of three independent ones

**Fig. 4:**
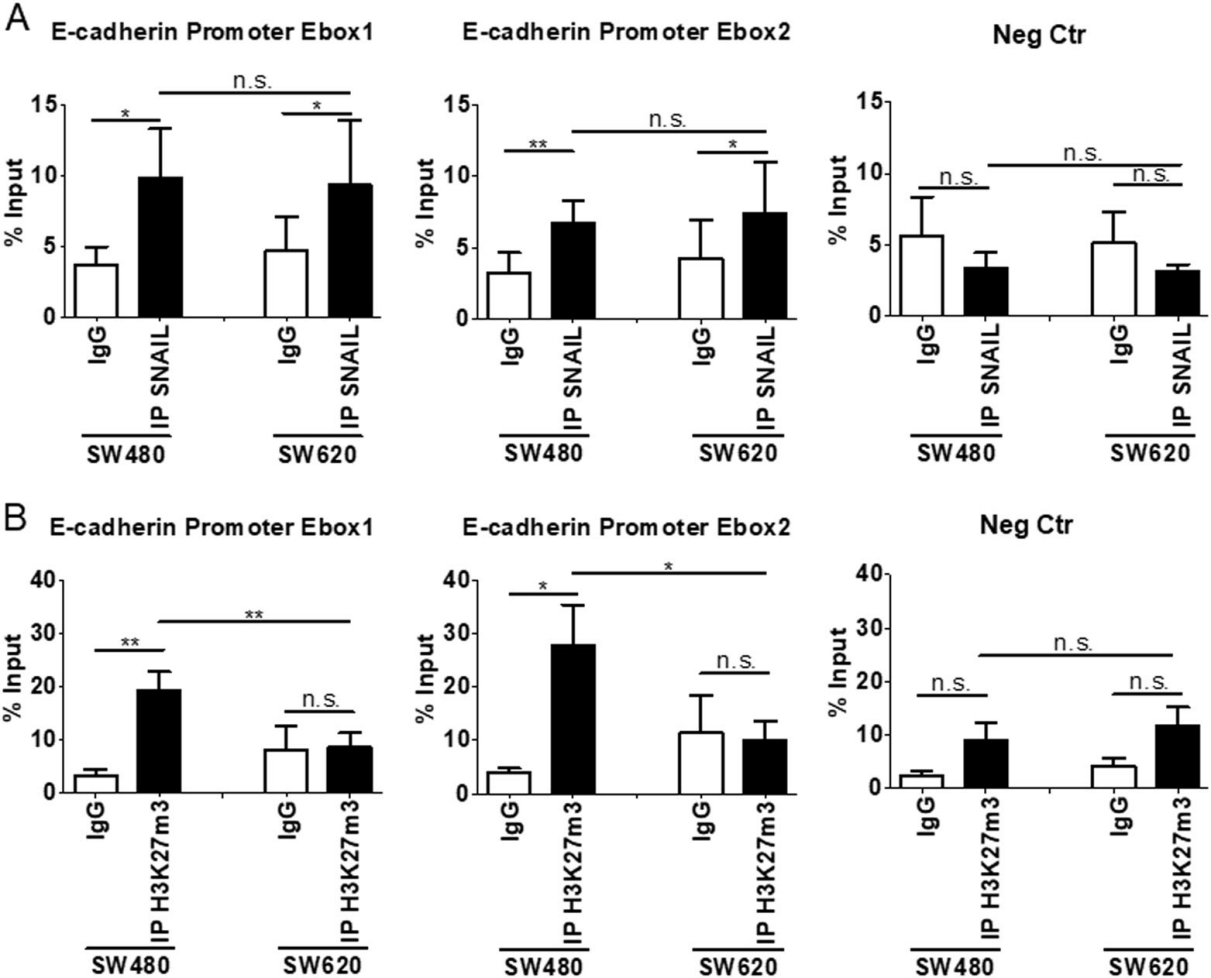
Snail function in colon cells. **a** qPCR analysis of ChIP assays with anti-Snail antibody (IP Snail) and, as controls, normal rabbit IgG (IgG) on chromatin from SW480 and SW620 cells. Data show the direct recruitment of endogenous Snail on the correspondent consensus binding sites (E-boxes) on the human *ECAD* promoter. Rpl30 sequences are analyzed as negative control (Neg Ctr). Values derived from five independent experiments are reported as means ± S.E.M. and expressed as percentage of the Input chromatin (% Input). Statistically significant differences (*p < 0.05; **p < 0.01; n.s. no significant) are reported. **b** qPCR analysis of ChIP assays with anti-H3K27me3 antibody (IPH3K27me3) and, as controls, normal rabbit IgG (IgG) on chromatin from SW480 and SW620 cells. Data show the enrichment of H3K27 trimethylation on the Snail consensus binding sites (E-boxes) on the human *ECAD* promoter. Rpl30 sequences are analyzed as negative control (Neg Ctr). Values derived from five independent experiments are reported as means ± S.E.M. and expressed as percentage of the Input chromatin (% Input). Statistically significant differences (*p < 0.05; **p < 0.01; n.s. no significant) are reported

To extend previous studies on the role of HOTAIR in the Snail-mediated repression of epithelial genes, we focused on the in vitro model of colon carcinoma progression represented by SW480 and SW620 cell lines, established, respectively, from a primary adenocarcinoma of the colon and from a secondary tumor from the same patient [28]. HOTAIR levels were analyzed in correlation with the expression of epithelial and mesenchymal genes known to be either causal or instrumental to the EMT, as previously reported in hepatocytes [10]. Data in Fig. 1 show that SW480 cells display an EMT molecular phenotype with expression of *Snail*, induction of the mesenchymal genes *metalloprotease 2 (MMP2*)*, vimentin* (*VIM*), and *fibronectin* (*FN1*), and negative regulation of the epithelial (and Snail-target) genes *E-cadherin* (*ECAD), HNF1A,* and *HNF4A* (Fig. 1a, b). With respect to HNF4A, these data are in line with previous observation, indicating that the loss of HNF4α plays a causal role in CRC progression [35]. On the contrary, the SW620 metastatic cells express *Snail* but also epithelial markers, thus suggesting an impairment of Snail repressive function (Fig. 1a, b). Notably, HOTAIR levels were high in SW480 and low in SW620 cells (Fig. 1a). Moreover, SW620 cells responded to the EMT inducer TGFβ with the downregulation of epithelial genes and with an increase of HOTAIR expression (Fig. 2a, b). These observations prompted us to hypothesize a causal role for HOTAIR in the regulation of Snail activity. Thus, we analyzed the response of the SW480 colon-derived cell line to HOTAIR silencing. As shown in Fig. 3a, b, HOTAIR knockdown in Snail-positive cells correlated with the impairment of Snail repressive activity on epithelial genes expression (i.e., *ECAD, HNF1A, HNF4A*). ChIP assays were then performed to investigate both Snail occupancy and the local H3K27me3 status of epithelial promoters. Snail was found bound to its consensus binding sites (E-boxes) on *ECAD* promoter in both cell lines (Fig. 4a) but the expected H3K27 trimethylation was impaired in SW620 cells (Fig. 4b), in which endogenous HOTAIR was downregulated (Fig. 1a). These data are in accord to previous results in hepatocytes [10]. Overall, these data provide further insights into HOTAIR function in colon carcinoma cells showing that this lncRNA (i) behaves as a mesenchymal gene in EMT and *(*ii) has a role in the control of Snail repressive activity.

### HNF4α directly inhibits HOTAIR expression

The evidence of HOTAIR induction in EMT and tumor progression [4, 11] and its causal role for epithelial gene repression (previously demonstrated in hepatocytes [10] and, here, in colon cells) led us to investigate the transcriptional control of HOTAIR gene.

**Fig. 5:**
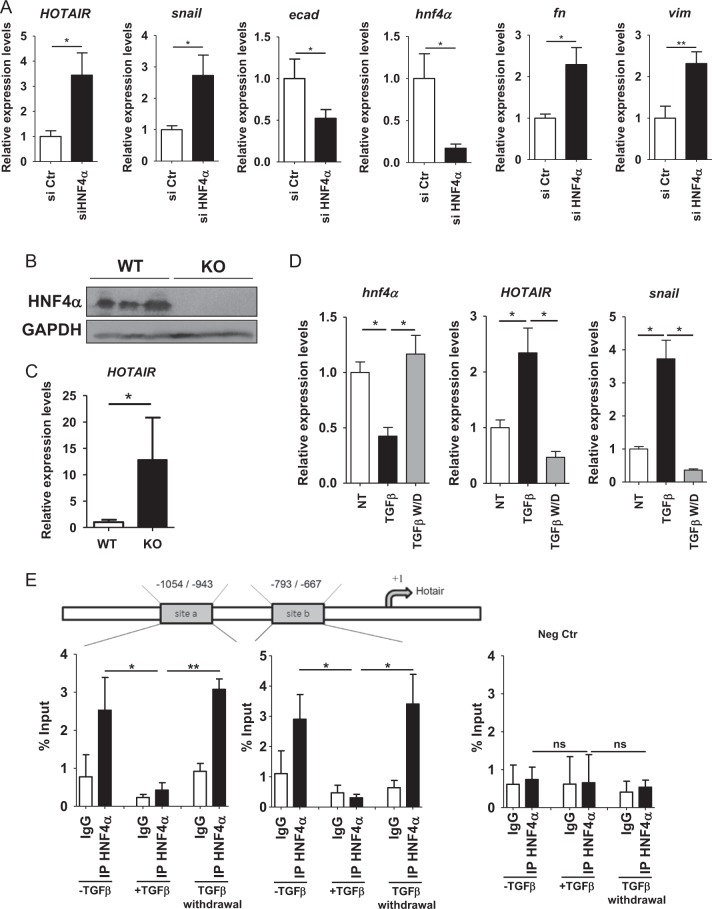
HNF4α regulates HOTAIR expression in hepatocyte. **a** RT-qPCR analysis for the indicated epithelial and mesenchymal markers on HNF4α-silenced (for 48 h) (siHNF4α) cells, compared with control siGFP cells (siCtr). The values are calculated by the ΔCt method, expressed as fold of expression vs. the control (arbitrary value = 1) and shown as means ± S.E.M. Statistically significant differences are reported (*p < 0.05; **p < 0.01) for five independent experiments. **b** Western blot analysis for HNF4α on protein extracts from liver samples from three hepatocyte-specific HNF4α KO mice and three matched Cre-negative littermates. Protein amount was normalized by immunoblotting for GAPDH, as indicated. **c** RT-qPCR analysis for HOTAIR on liver samples from six hepatocyte-specific HNF4α KO mice and five matched Cre-negative littermates. The values are calculated by the ΔCt method, expressed as fold of expression vs. the control (arbitrary value = 1) and shown as means ± S.E.M. Statistically significant differences are reported (*p < 0.05). **d** RT-qPCR analysis for the indicated markers on hepatocytes treated (TGFβ) or not (NT) with TGFβ and after cytokine withdrawal (TGFβ W/D). The values are calculated by the ΔCt method, expressed as fold of expression vs. the control (arbitrary value = 1) and shown as means ± S.E.M. Statistically significant differences are reported (*p < 0.05) for three independent experiments. **e** qPCR analysis of ChIP assays with an anti-HNF4α antibody, or normal rabbit IgG as negative control, on chromatin from TGFβ-treated cells (+ TGFβ) or controls (− TGFβ) for 24 h and after TGFβ withdrawal, showing endogenous HNF4α binding on HOTAIR promoter consensus − 1054/− 943 (site a) and −793/−667 (site b). Timm promoter sequences were analyzed as control (Neg Ctr). Values derived from five independent experiments are reported as means ± S.E.M. and expressed as percentage of the Input chromatin (% Input). Statistically significant differences (*p < 0.05; **p < 0.01; n.s. no significant) are reported

Previous researches highlighted that: (i) the master factor HNF4α directly represses several master EMT regulators and mesenchymal genes (e.g., *SNAI1, SNAI2, HMGA2, FN1, VIM*) both in MET and in the stable maintenance of the epithelial identity [22]; (ii) HNF4α impairment, occurring at the transcriptional and posttranslational levels, is causal to EMT [22, 23]; (iii) Snail, in turn, is a direct repressor of HNF4α [36]. Given this body of evidence, we investigated whether the molecular mechanisms by which HNF4α antagonizes EMT include the direct repression of HOTAIR. To this aim, we first focused on hepatocytes and monitored HOTAIR expression in HNF4α-silenced cells. Once confirmed (in line with ref. [22]) that HNF4α silencing induced both the master regulator Snail and the mesenchymal markers fibronectin and vimentin and caused the repression of the epithelial marker E-cadherin, we measured HOTAIR levels in the same HNF4α-interfered cells and found this lncRNA upregulated (Fig. 5a). Our observations were also extended to an in vivo model, by using hepatocyte-specific *Hnf4a*-null mice (*Hnf4a*^*F/F;AlbERT2cre*^ [27]) and matched *Hnf4a*^*F/F*^ littermates (Fig. 5b), previously shown to display a marked induction of Snail and of various mesenchymal products [22]. HOTAIR, undetectable in the *Hnf4a*^*F/F*^ controls, was found strongly induced in *Hnf4α*^*F/F; AlbERT2cre*^ hepatocytes (Fig. 5c). Notably, HOTAIR expression paralleled that of mesenchymal genes also during MET: HOTAIR expression was upregulated in TGFβ-mediated EMT and downregulated following TGFβ withdrawal, when cells restored the epithelial phenotype and reverted the EMT-related gene expression (as demonstrated for HNF4α and Snail; Fig. 5d and [22]). Sequence inspection revealed the presence of two putative HNF4α binding sites on the murine HOTAIR promoter. Therefore, to address the hypothesis that HNF4α could directly control the expression of this lncRNA, ChIP assays were performed. As shown in Fig. 5e, ChIP analysis demonstrated the recruitment of endogenous HNF4α to the promoter of HOTAIR in hepatocytes and its displacement during EMT (when HNF4α is negatively regulated by TGFβ and HOTAIR is induced [10, 23, 24]). Furthermore, the MET occurring after TGFβ withdrawal correlated with HNF4α re-binding on the HOTAIR regulatory sequences (Fig. 5e). We next hypothesized that HNF4α could physically regulate HOTAIR also in colon cells, consistent with both the well-known role of HNF4α in epithelial colon cells differentiation [17] and the above-described functional role of HOTAIR in EMT of colon cells (Figs. 1–3).

**Fig. 6:**
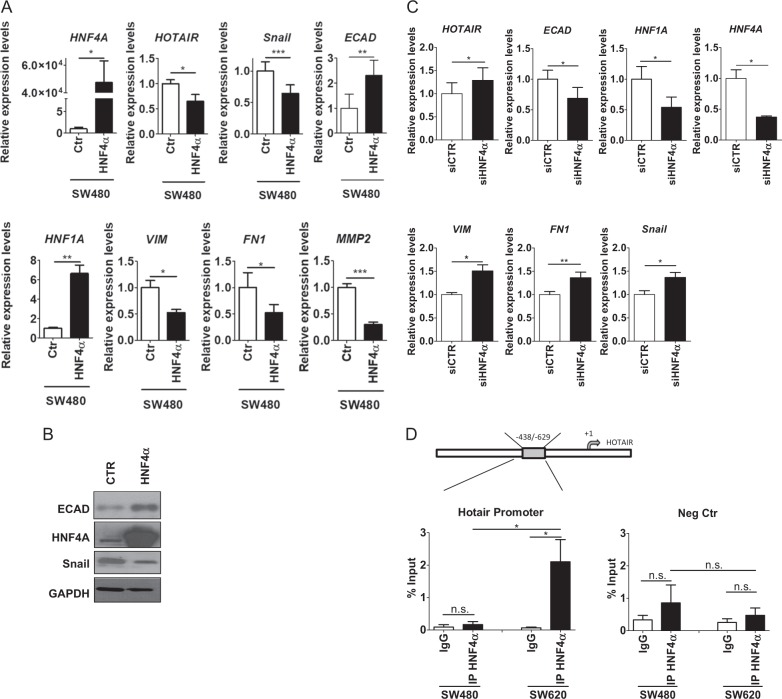
HNF4α regulates HOTAIR expression in colon cells. **a** RT-qPCR analysis for the indicated markers on SW480 cells overexpressing HNF4α (HNF4α), compared with mock-infected control cells (Ctr). The values are calculated by the ΔCt method, expressed as fold of expression vs. the control (arbitrary value = 1) and shown as means ± S.E.M. Statistically significant differences are reported. Statistically significant differences (*p < 0.05; **p < 0.01; ***p < 0.001) are reported for five independent experiments. **b** Western blot analysis for the indicated markers on protein extracts from cells treated as in **a**. Protein amount was normalized by immunoblotting for GAPDH, as indicated. All the experiments have been performed in triplicate and WB images represent one indicative experiment of three independent ones. **c** RT-qPCR analysis as in **a** on HNF4α-silenced-SW620 cells (siHNF4α) in comparison with siGFP cells (siCtr), as control. Statistically significant differences (*p < 0.05; **p < 0.01) are reported for four independent experiments. **d** qPCR analysis of ChIP assays with anti-HNF4α antibody (IP HNF4α) and, as control, normal rabbit IgG (IgG) on chromatin from SW480 and SW620 cells. Data show the direct recruitment of endogenous HNF4α on the correspondent consensus binding site on the human promoter of HOTAIR. *Rpl30* promoter was used as a negative control. Values derived from five independent experiments are reported as means ± S.E.M. and expressed as percentage of the Input chromatin (% Input). Statistically significant differences (*p < 0.05; n.s. no significant) are reported

To address the question, we overexpressed HNF4α in SW480 cells (that lack endogenous HNF4α and display both HOTAIR expression and a EMT phenotype; Fig. 1a, b). As shown in Fig. 6a, expression of ectopic HNF4α in SW480 cells triggered a MET with upregulation of *ECAD* and *HNF1A* mRNA, negative regulation of the mesenchymal markers *VIM, FN1*, and *MMP2* mRNAs and of the master factor *SNAI1* mRNA (Fig. 6a, b); remarkably, in this condition HOTAIR expression was downregulated (Fig. 6a). Conversely, HNF4α silencing in SW620 cells (that express HNF4α and display an epithelial phenotype; Fig. 1a, b) resulted in the loss of epithelial markers and the induction of the mesenchymal ones, including HOTAIR (Fig. 6c). These results were in line with the inverse correlation between HNF4α and HOTAIR levels observed in SW480 and SW620 cells (Fig. 1).

As a bioinformatic search by MatInspector analysis highlighted putative HNF4α consensus sites also on the human HOTAIR promoter, ChIP assays were performed on chromatin from both cells lines. As shown in Fig. 6d, endogenous HNF4α binding was demonstrated in SW620 cells, whereas absent in EMT-like SW480 cells.

Overall, these data indicate that HOTAIR is directly repressed by HNF4α, both in the stable maintenance of epithelial identity and during MET transdifferentiation, whereas HOTAIR gene repression is released by HNF4α impairment, both in hepatocytes and in colon carcinoma cells.

### The direct inhibition of HOTAIR expression by HNF4α correlates with the release of a chromatin loop

**Fig. 7:**
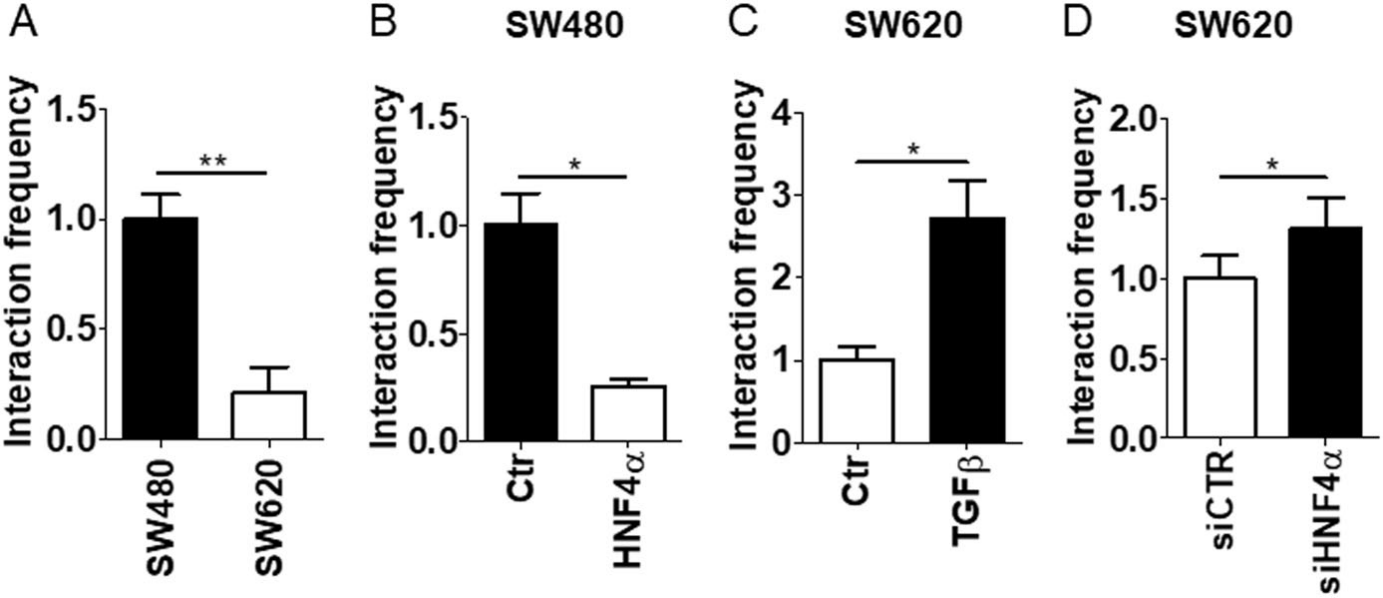
HNF4α binding to HOTAIR regulative sequences associates to chromatin topological changes **a** 3C assays of the HDE-HOTAIR locus in SW480 and SW620 cells. Values derived from three independent experiments are reported as means ± S.E.M. and expressed with respect to the control sample. Statistically significant differences are reported (**p < 0.01). **b** 3C assay of the HDE-HOTAIR locus in HNF4α- or mock-infected SW480 cells. Values derived from three independent experiments are reported as means ± S.E.M. and expressed with respect to the control sample. Statistically significant differences are reported (*p < 0.05). **c** 3C assay of the HDE-HOTAIR locus in SW620 cells treated or not with TGFβ. Values derived from three independent experiments are reported as means ± S.E.M. and expressed with respect to the control sample. Statistically significant differences are reported (*p < 0.05). **d** 3 C assay of the HDE-HOTAIR locus in HNF4α-silenced (for 48 h) (siHNF4α) SW620 cells, compared with control siGFP cells (siCtr). Values derived from three independent experiments are reported as means ± S.E.M. and expressed with respect to the control sample. Statistically significant differences are reported (*p < 0.05)

One of the few-reported observations on HOTAIR transcriptional regulation includes characterization in human breast cancer cells of an enhancer, termed HOXC Distal Enhancer (HDE) located 150 Kb downstream of the HOTAIR TSS [14]. The HDE was proven to engage in long-range interactions with the HOTAIR promoter to establish a chromatin loop functional to positively regulate the transcription [14]. We therefore hypothesized a causal relationship between the HNF4α binding to the HOTAIR promoter and topological remodeling of this three-dimensional structure. To validate this hypothesis, we performed chromosome conformation capture (3 C) assays: cross-linked chromatins, extracted in different cell conditions, were subjected to digestion and ligation, then qPCR reactions were performed to amplify the fragments obtained from the ligation of the HDE sequences to HOTAIR proximal promoter. Data shown in Fig. 7 demonstrated that although the two regions are associated when HOTAIR expression is positively regulated, this association is disrupted when HNF4α is recruited to the HOTAIR promoter, allowing repression of transcription. This conclusion was confirmed under different conditions i.e.: (i) SW480 cells, that lack endogenous HNF4α, in comparison with the MET-like SW620 cells (Fig. 7a); (ii) SW480 cells, in comparison with the same cells ectopically expressing HNF4α (Fig. 7b); (iii) SW620 cells treated or not treated with TGFβ, whose mediated EMT causes the HNF4α displacement from its binding site ([23], Fig. 7c); (iv) finally, the causal relationship between HNF4α binding and disruption of the three-dimensional chromatin loop was proven in SW620 cells by HNF4α knockdown (Fig. 7d), above reported to induce HOTAIR expression (Fig. 6c).

## Discussion

**Fig. 8:**
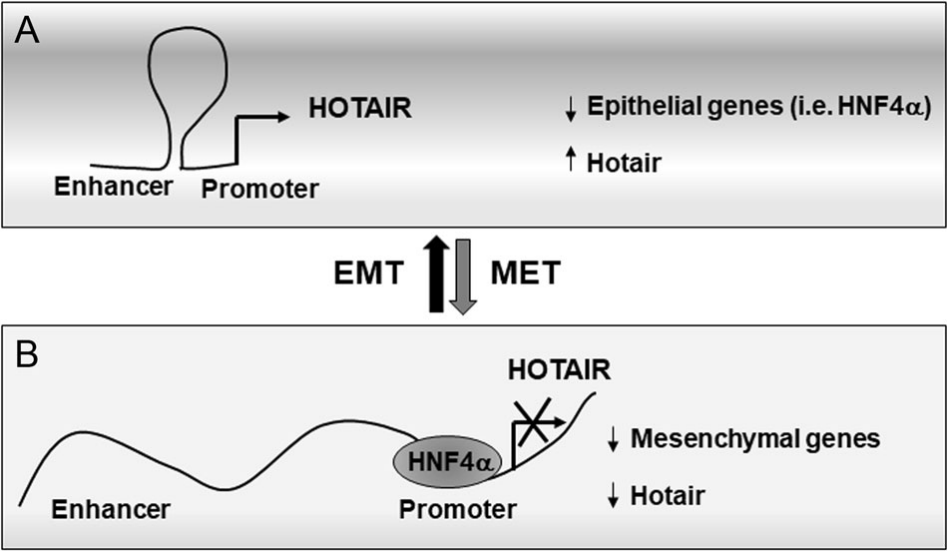
Scheme of the proposed mechanism of HOTAIR regulation. **a** In EMT cells HNF4α is negatively regulated and HOTAIR expression positively controlled by the enhancer. **b** In epithelial cells HNF4α represses HOTAIR transcription by interfering with the formation of a regulatory chromatin loop

The main finding of this study is the identification of a molecular mechanism controlling the expression of the oncogene HOTAIR. HNF4α, a master factor of MET and inducer of epithelial differentiation, was found to directly repress HOTAIR transcription thus antagonizing the EMT of both hepatocytes and colon cancer cells. Mechanistically, our data revealed an enhancer-blocking activity of HNF4α by influencing chromatin topology of HOTAIR gene regulatory sequences (Figs. 7 and 8).

Despite the proven oncogenic role of HOTAIR, its regulation is still poorly understood. HOTAIR is a low-copy lncRNA [37], which expression is correlated with TGFβ-mediated EMT [10, 38] and clinical prognosis in different tumor types (i.e., breast [2], colorectal ([6]), nasopharyngeal [13], and in liver cancer [4, 11, 12, 31]. In fact, higher levels of HOTAIR have been shown to promote cancer metastasis by modulation of PRC2-specific binding to chromatin, thus reprogramming the cell state to resemble the fibroblast condition [2]. We recently showed that the master EMT factor SNAI1 requires the direct enrollment of HOTAIR, in turn scaffolding EZH2, to direct the Polycomb catalytic member to targets pivotal in epithelial morphogenesis and differentiation (i.e., HNF4α, HNF1α, and ECAD). Here, we extended this role of HOTAIR to colon carcinoma cells conferring a more general value to the mechanism of how EZH2 gets to its genomic targets in epithelial cells undergoing EMT. ChIP experiments provided evidence that SNAI1 occupancy on target promoters is independent from HOTAIR, whereas Snail repressive activity, and the related modifications of chromatin marks guiding the EMT, requires HOTAIR.

HNF4α is a well-known (i) master factor of epithelial cell differentiation [15–17, 39]; (ii) master factor of epithelial identity maintenance [22]; as well as (iii) a MET inducer [15, 22, 38], by acting as an activator as well as a transcriptional repressor. Its role of tumor suppressor is underlined by the fact that HNF4α loss is determinant for both HCC CRC onset and progression [35, 15]. Notably, the master role of HNF4α implies its stable active repression of several mesenchymal genes (i.e., SNAI1, SNAI2, HMGA2, VIM, and FN1) [22]. Here, the HNF4α repressive activity is extended to HOTAIR transcription both in hepatocytes and colon cancer cells. HNF4α silencing induces HOTAIR expression both in vitro and in the hepatocyte-specific *Hnf4a*-null mouse model, and HOTAIR induction pairs the HNF4α functional impairment in the TGFβ-induced EMT. Moreover, ChIP analysis revealed that HNF4α directly binds to the HOTAIR promoter and its binding inversely correlates with HOTAIR transcription. The inverse correlation between HOTAIR and HNF4α expression was confirmed in colon cancer cells, with HOTAIR upregulation in EMT-like SW480 cells, expressing low levels of HNF4α, and HOTAIR downregulation in MET-like HNF4α-positive SW620 cells. Moreover, this inverse correlation was observed also in SW480 cells during HNF4α-induced MET and in SW620 undergoing a TGFβ-mediated EMT or after HNF4α silencing. The ChIP data suggest that in this cell type, HNF4α directly binds to the HOTAIR promoter and regulates HOTAIR gene repression.

Furthermore, our results integrate the well-established knowledge about the role of HNF4α as a transcriptional repressor with a new function, attributing to this factor an enhancer-blocking activity. Others previously reported on a HOTAIR enhancer able to positively regulate HOTAIR gene expression by DNA looping [14]. Here, by means of the 3 C technique [40, 41], we investigated how structural interactions between these regulatory elements relates to HOTAIR gene expression in presence or absence of HNF4α. Provided results demonstrated that HNF4α recruitment to HOTAIR promoter disrupts the ability of the enhancer to contact HOTAIR promoter elements, resulting in the gene repression. The robustness of this conclusion was confirmed in SW480 and SW620 cells grown in basal culture conditions as well as by evaluating the effects of HNF4α overexpression, its silencing or impairment of its activity after TGFβ-mediated treatment.

Our data should be considered in line with other dynamic changes of higher-order chromatin structures, previously described for specific loci during differentiation [42, 43] or in cancer [44, 45]. Furthermore, this evidence opens new perspectives into the mechanisms of how a master regulatory factor acts in the coordinate regulation of several targets. It is conceivable, indeed, that the ability of HNF4α to cause spatial reorganization of chromatin might be applied to other genes and linked to the capacity to recruit chromatin modifying complexes in specific sites.

## Electronic supplementary material

Supplemental Table 1
