# Supplementary material for: The lncRNA HOTAIR transcription is controlled by HNF4α-induced chromatin topology modulation
Source: Cell Death Differ. 2018 Aug 28;26(5):890–901. doi: 10.1038/s41418-018-0170-z (PMC6461983; doi:10.1038/s41418-018-0170-z)
Supplement: Supplementary file 1 — Supplemental Table 1 [file 41418_2018_170_MOESM1_ESM.docx]

| **Supplemental Material:**  **Table 1. List of primers** | |
| --- | --- |
| ***Oligonucleotides for gene expression analysis*** | |
| ***Target gene*** | ***Primer sequences (5′-3′)*** |
| *snail (mus musculus)*  *hnf4α (mus musculus)*  *e-cadherin (mus musculus)*  *l34 (mus musculus)*  *18S (mus musculus)*  *hotair (mus musculus)*  *vimentin (mus musculus)*  *fibronectin (mus musculus)*  *HOTAIR (homo sapiens)*  *L32 (homo sapiens)*  *HNF4α (homo sapiens)*  *Snail (homo sapiens)*  *E-cadherin (homo sapiens)*  *MMP2 (homo sapiens)*  *Fibronectin (homo sapiens)*  *HNF1α (homo sapiens)*  *Vimentin (homo sapiens)* | Fw, CCACTGCAACCGTGCTTTT Rev, CACATCCGAGTGGGTTTGG  Fw, TCTTCTTTGATCCAGATGCC Rev, GGTCGTTGATGTAATCCTCC  Fw, CTACTGTTTCTACGGAGGAG Rev, CTCAAATCAAAGTCCTGGTC  Fw, GGAGCCCCATCCAGACTC Rev, CGCTGGATATGGCTTTCCTA  Fw, ACGACCCATTCGAACGTCTG Rev, GCACGGCGACTACCATCG  Fw, GCGCCAACGTAGACCAAAAG Rev, TACCGATGTTGGGGACCTCT  Fw, AGCAGTATGAAAGCGTGGCT  Rev, CTCCAGGGACTCGTTAGTGC  Fw, AGACCATACCTGCCGAATGTAG  Rev, GAGAGCTTCCTGTCCTGTAGAG  Fw, CGGGACTTAGACCCTCAGGT Rev, GTTCCATTCCACTGCGAAGC  Fw, GGAGCGACTGCTACGGAAG  Rev, GATACTGTCCAAAAGGCTGGAA  Fw, CATGGACATGGCCGACTACA  Rev, ATTGCCCATCGTCAACACCT  Fw, CACTATGCCGCGCTCTTTC  Rev, GCTGGAAGGTAAACTCTGGATTAGA  Fw, TACGCCTGGGACTCCACCTA  Rev, CCAGAAACGGAGGCCTGAT  Fw, ATGCCGCCTTTAACTGGAG  Rev GGAAAGCCAGGATCCATTTT  Fw, GGCTGACAGAGAAGATTCCCG  Rev AGCTGGGTCTGCTAACATCAC  Fw, GCCCACCAAGCAGGTCTTCA  Rev AGGGTCCTGGCTGGGGAC  Fw, GCTAACCAACGACAAAGCCC  Rev, GATTGCAGGGTGTTTTCGGC |
| ***Oligonucleotides for ChIP analysis*** | |
| ***Target gene*** | ***Primer sequences (5′-3′)*** |
| *HOTAIR Promoter (site a) (mus musculus)*  *HOTAIR Promoter (site b) (mus musculus)*  *Negative Ctr (mus musculus)*  *E-caderin Promoter (E-box 1)*  *(homo sapiens)*  *E-caderin Promoter (E-box 2)*  *(homo sapiens)*  *HOTAIR Promoter (homo sapiens)*  *Negative Ctr (homo sapiens)* | Fw, CCCTCATCCCCTACACAAAG  Rev, GGAATCCTGGTTTCTCCACA  Fw, TTCCCAGCCAGGTAGGTAGA  Rev, TCAGGAGTGATGATGGGTGA  Fw, ACGGATGTGGCCCTTCTGGCT  Rev, CCGCTCCGAAACGCCCACAA  Fw, GGCAAGACAGAGCGAGAC  Rev, TCGAACTCCTGGGCTGAA  Fw, GGTGAACCCTCAGCCAATCA  Rev, CACAGGTGCTTTGCAGTTCC  Fw, TAGAGGGTCCAGGAGGTTCC  Rev, CGGGAAATCCTCTGCTGTAA  Fw, GCAGGAAGATGGTGGCCGCAA  Rev, AGTCTGCTTGTACCCCAGGACGT |
| ***Oligonucleotides for 3C assays analysis*** | |
| ***Target gene*** | ***Primer sequences (5′-3′)*** |
| *GAPDH Promoter*  *hoxc Enhancer*  *HOTAIR Gene* | Fw, TACTAGCGGTTTTACGGGCG  Rev, TCGAACAGGAGGAGCAGAGAGCGA  Fw, CCTGGGTGGAGGAGTGAGAAGACATACC  Rev, GAGCTCTGCTGCTTGCAGTGGAATGG  Fw, CAGGCGATAAATCTCTGGCGAATCTTCC  Rev, TTCGGAACCTCCTTTTCCGAGCAGC |
